# Vertebrates originally possess four functional subtypes of G protein-coupled melatonin receptor

**DOI:** 10.1038/s41598-019-45925-2

**Published:** 2019-07-01

**Authors:** Kotowa Sakai, Yuya Yamamoto, Toshitaka Ikeuchi

**Affiliations:** Graduate School of Biosciences, Nagahama Institute of Bio-Science and Technology, 1266, Tamura, Nagahama, Shiga 526-0829 Japan

**Keywords:** Zoology, Evolution, Zoology, Evolution

## Abstract

Melatonin receptors (MTNRs) belonging to the G protein-coupled receptor family are considered to consist of three subtypes in vertebrates: MTNR1a, MTNR1b and MTNR1c. Additionally, *MTNR1a-like* genes have been identified in teleostean species as a fish-specific subtype of MTNR1a. However, similar molecules to this *MTNR1a-like* gene can be found in some reptiles upon searching the DNA database. We hypothesized that a vertebrate can essentially have four functional subtypes of MTNR as ohnologs. Thus, in the present study we examined the molecular phylogeny, expression patterns and pharmacological profile(s) using the teleost medaka (*Oryzias latipes*). The four conserved subtypes of MTNR (MTNR1a, MTNR1b, MTNR1c and MTNR1a-like) in vertebrates were classified based on synteny and phylogenetic analysis. The fourth MTNR, termed MTNR1a-like, could be classified as MTNR1d. It was observed by using RT-qPCR that expression patterns differed amongst these subtypes. Moreover, *mtnr1a*, *mtnr1c* and *mtnr1a-like*/*mtnr1d* expression was elevated during short days compared to long days in diencephalons. All the subtypes were activated by melatonin and transduced signals into the Gi pathway, to perform a cAMP-responsive reporter gene assay. It was shown that MTNR originally consisted of four subtypes: MTNR1a, MTNR1b, MTNR1c and MTNR1d. These subtypes were functional, at least in fish, although some organisms, including mammals, have lost one or two subtypes.

## Introduction

Melatonin (N-acetyl-5-methoxytryptamine) is primarily synthesized in the pineal gland and retina in all vertebrate species analyzed, and is considered a potent regulator of circadian and seasonal rhythms^1^. Melatonin exists in almost all living organisms, and its actions are mediated by binding to G protein-coupled receptors (GPCR)^2^, nuclear receptors^3^, or a low affinity protein (quinone reductase 2; QR2)^4^. However, the observation concerning nuclear receptors has been challenged^5^, and the homolog in the medaka did not display efficiently promoted transcriptional activity by melatonin^6^. The GPCR types are named MTNRs and are divided into three subtypes, MTNR1a (MT1/Mel1a), MTNR1b (MT2/Mel1b) and MTNR1c (Mel1c). MTNR1a and MTNR1b were identified in almost vertebrate species, whereas MTNR1c was only found in non-mammalian species^2^. Additionally, MTNR1a (MTNR1a1/MTNR1a1.7) and MTNR1a-like (MTNR1a2/MTNR1a1.4) have been identified as two distinct subtypes of MTNR1a in teleostean species^7–11^. Many gene families in vertebrates originated during two rounds of whole genome duplication occurring early in the evolution of the Chordata^12–14^. An additional fish-specific whole genome duplication (FSGD) occurred in the teleostean lineage^15^. Applying *MTNR* genes to this scenario, the four paralogs *MTNR1a*, *MTNR1b*, *MTNR1c* and ‘*MTNR1d*’ were thought to have been generated after the second genome duplication event. Because mammals possess only *MTNR1a* and *MTNR1b*, they must have lost *MTNR1c* and *MTNR1d*. In non-mammalians, *MTNR1d* was lost and *MTNR1a*, *MTNR1b* and *MTNR1c* remained. Interestingly, there are four subtypes, as within teleosts and some reptiles, which did not undergo teleost-specific whole genome duplication. It is possible that the *MTNR1a-like* gene is not a fish-specific paralog derived from FSGD, but is equivalent to *MTNR1d*. Because the above-mentioned classification for the MTNR subtypes appears contradictory, we attempted to investigate MTNR evolutionary history using molecular phylogenetic and syntenic relationships analyses in the current study.

Thus, a new question arises, if the MTNR1a-like (MTNR1a2/MTNR1a1.4) is the ortholog corresponding to the lost MTNR1d in mammals and birds, does MTNR1d remain as the receptor for melatonin? Divergence of genes often leads to neo-functionalization or non-functionalization^15^. There are no reports on whether MTNR1d (MTNR1a-like/MTNR1a2/MTNR1a1.4) can work as a GPCR following melatonin binding. Even MTNR1c exhibits no evidence indicating that it is the functional receptor in teleosts. Therefore, it is necessary to clarify that MTNRs are able to transduce melatonin signals.

Here, we try to investigate for the existence of four ‘functional’ subtypes in MTNR and their relationship(s).

## Results

### Synteny and phylogenetic analysis

**Figure 1:**
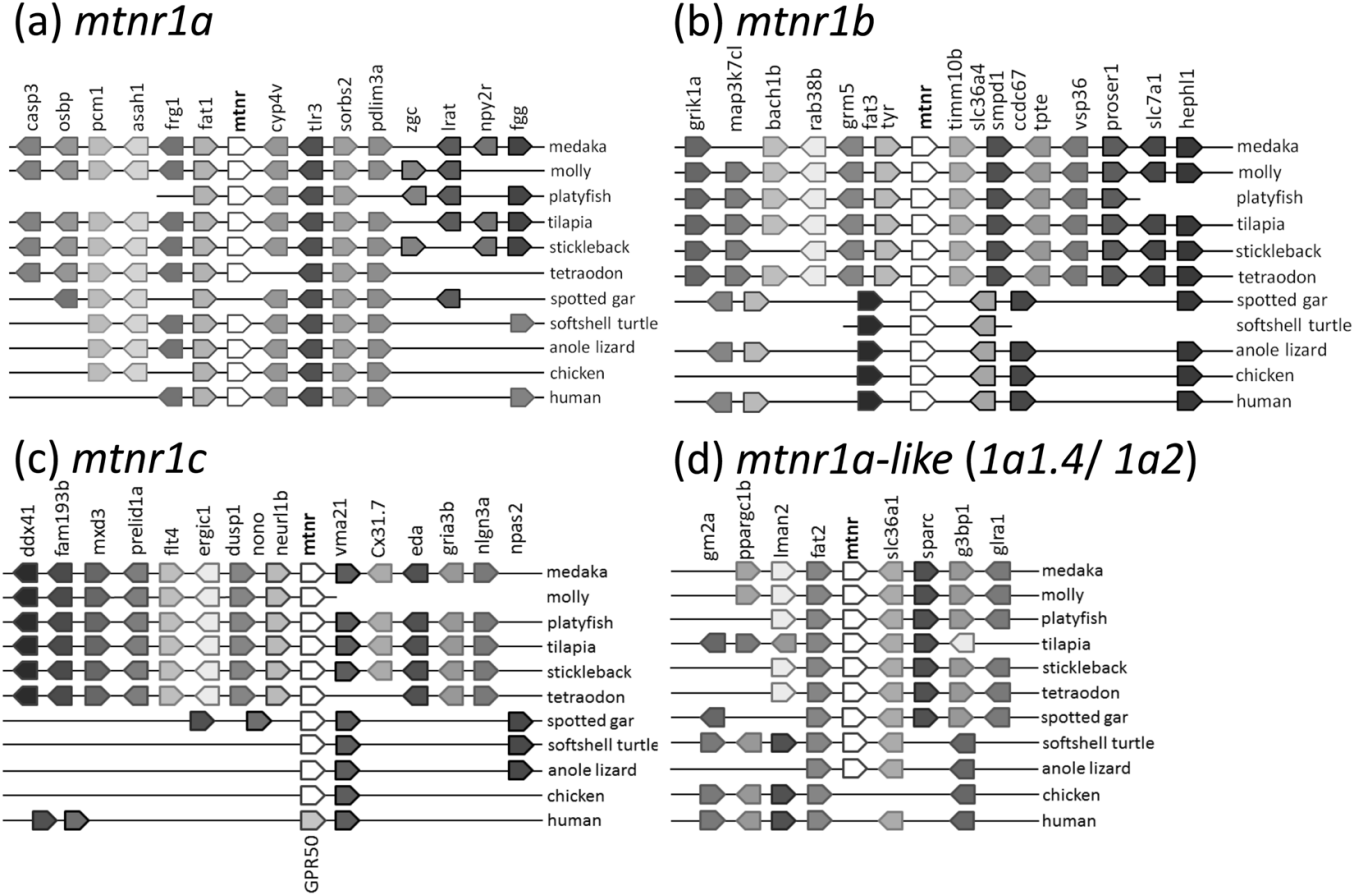
Patterns of synteny in the neighboring genes of *mtnr* in vertebrates. The syntenic region near each medaka subtype, *mtnr1a* (**a**) *mtnr1b* (**b**) *mtnr1c* (**c**) and *mtnr1a-like* (**d**), were compared among vertebrates. The medaka genome was used as a reference. Genes are represented by block arrow(s), equal brightness indicates ortholog genes and the tip of block arrows denote the direction of the gene.

Phylogenetic and synteny analyses were performed to make inferences regarding the occurrence of the *MTNR* paralogs conserved in vertebrates. In many vertebrates the *fat1* and *cyp4v* genes are situated adjacent to *mtnr1a*; however, the spotted gar has lost *mtnr1a* between these genes (Fig. 1a). The cluster of genes surrounding *mtnr1b* differs between teleosts and other vertebrates (Fig. 1b). However, the location of the *hephl1* gene is to the same in both teleosts and tetrapods. Although synteny of the *mtnr1c* gene also shows differences between teleosts and other vertebrates, *vma21* was located next to *mtnr1c* in many species (Fig. 1c). The gene residing next to *vma21* was not *mtnr1c*, but *gpr50* in humans. The pattern of synteny in genes neighboring the fourth subtype of the *mtnr* gene, named *mtnr1a-like*, was conserved in a form sandwiched between *fat2* and *slc36a1* (Fig. 1d). This form was similar in the spotted gar, softsell turtle and anole lizard. Chickens and humans do not have this fourth *mtnr* homolog, even though its synteny shares a close similarity to these holosteans and reptiles.

**Figure 2:**
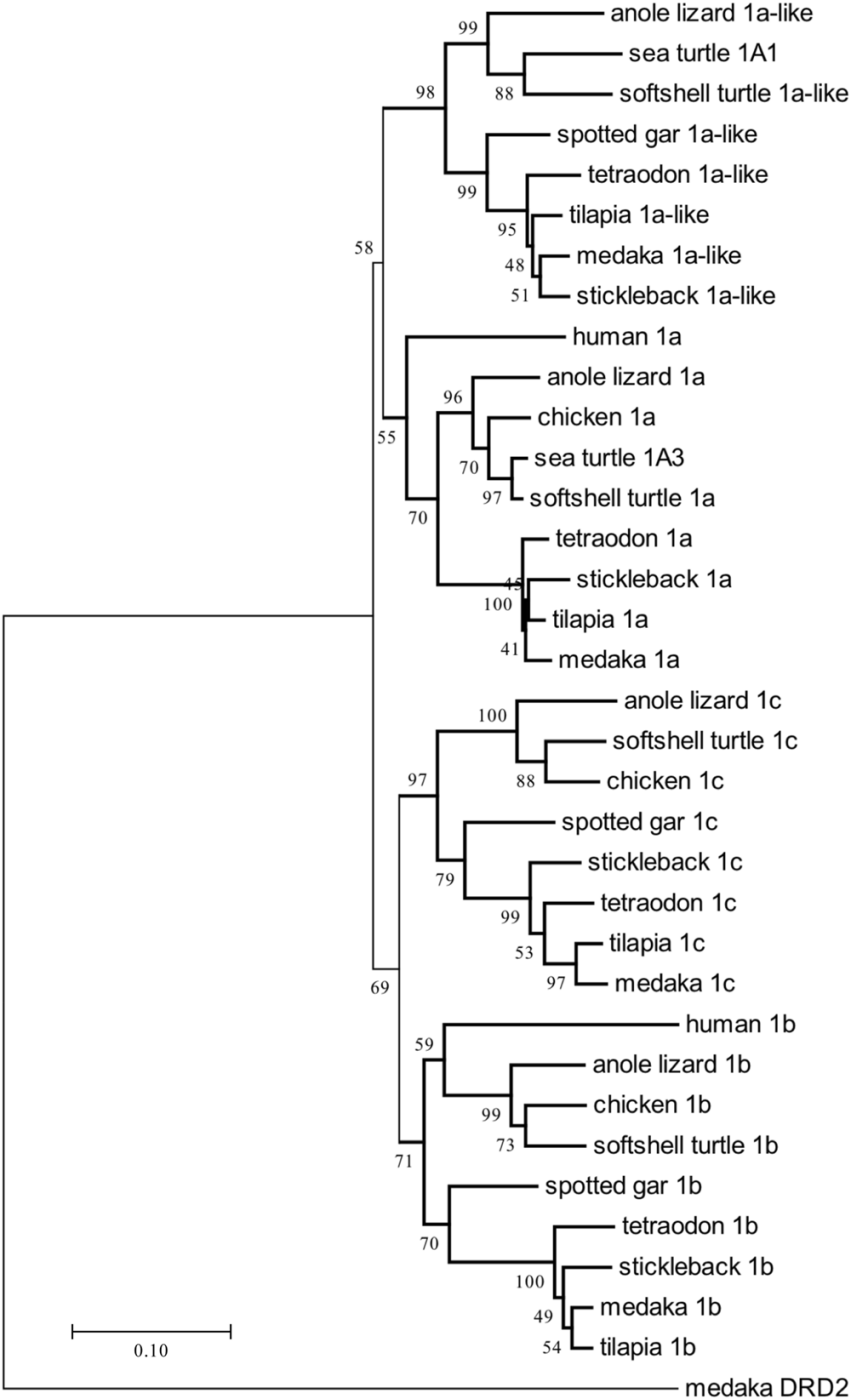
Phylogenetic tree based on the protein sequences of the MTNRs in vertebrates. Medaka DRD2 was used as an outgroup in the phylogeny. Amino acid sequences were aligned and a phylogenetic tree was constructed with the MEGA 7.0.14 software using the neighbor-joining method. Branch support values were estimated using bootstrap tests with 1000 replications, shown next to the branches.

By using phylogenetic analysis it was determined that the four groups of *MTNR* formed monophyletic clades (Fig. 2). These clades correspond with *MTNR1a* (*MT1*/*Mel1a*), *MTNR1b* (*MT2*/*Mel1b*), *MTNR1c* (*Mel1c*) and the fourth subtype, called *MTNR1a-like* (*MTNR1a2*/*MTNR1a1*.*4*). It is conceivable that these are ohnologs. Two groups, the ancestral subtype of *MTNR1a* and *MTNR1a-like* as well as the ancestral subtype of *MTNR1b* and *MTNR1c*, branched off from a common ancestor. Subsequently, four subtypes were derived from these. The *MTNR* of four teleostean fishes, the soft-shelled turtle and anole lizard presented in each group the four subtypes, and the two *MTNR1a*’s of the sea turtle *MTNR* belonged to the group of *MTNR1a* or *MTNR1a-like*, respectively.

### Melatonin-dependent regulation for cAMP-response reporter gene by MTNRs

**Figure 3:**
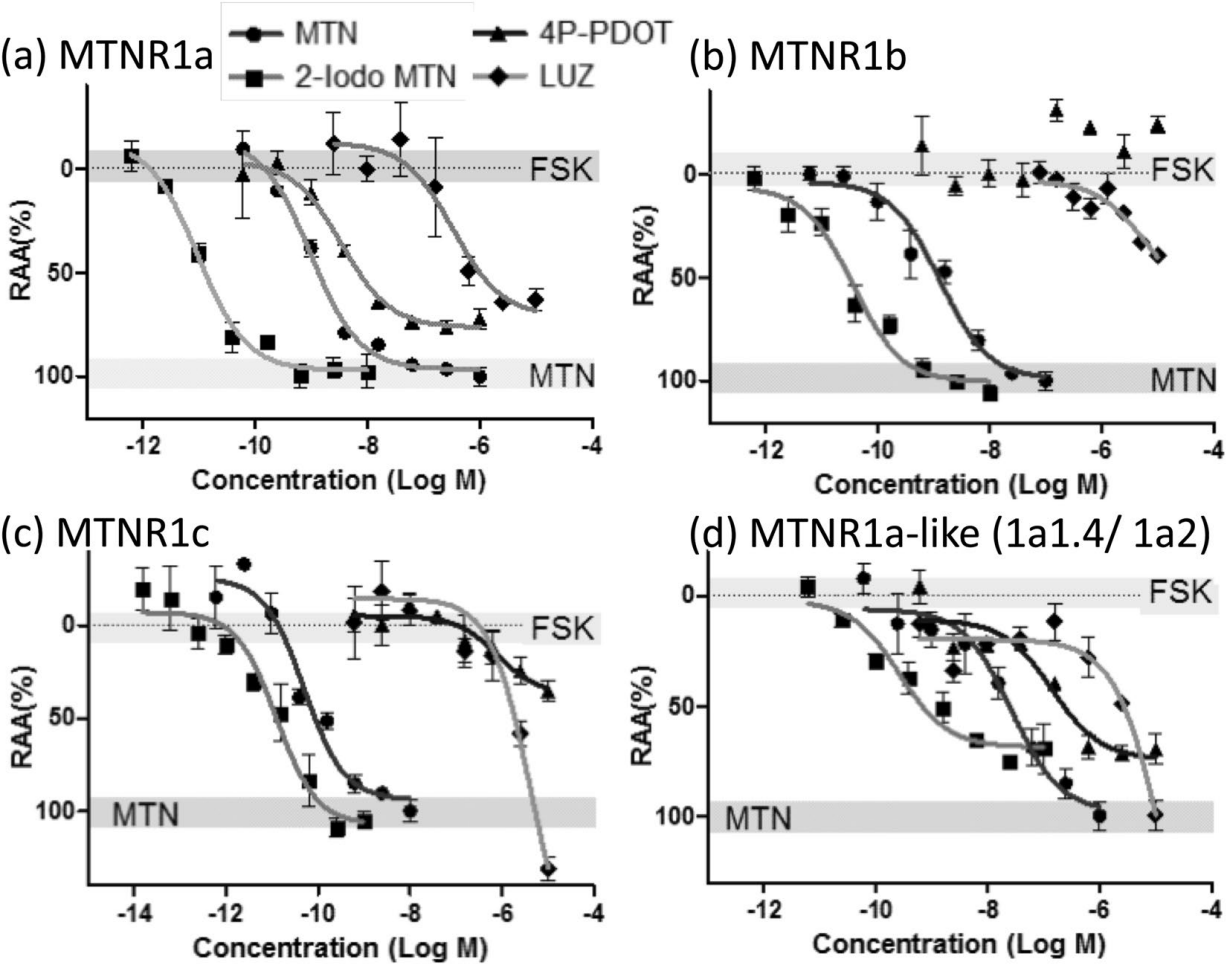
Concentration-response curve of typical MTNR ligands for medaka MTNRs. The y-axis shows the relative agonistic activity (RAA) and x-axis shows the concentration of reagents treated for cells. The upper gray box signifies the mean ± standard error of the mean (S.E.M.) value of forskolin (FSK) and the lower gray box represents the average (±S.E.M.) value of the maximum effect on melatonin (MTN). Each point represents the mean of triplicate determinations, and vertical bars represent the S.E.M.

All MTNRs expressed in cells are reduced cAMP-responsive reporter activity in a dose-dependent manner via melatonin (Fig. 3); however, their sensitivities differ. MTNR1c was the most sensitive for melatonin with an EC_50_ = 0.062 ± 0.006 nM. The second was MTNR1a with an EC_50_ of 0.48 ± 0.2 nM. The third and final were MTNR1b (EC_50_ = 1.9 ± 0.49 nM) and MTNR1d (EC_50_ = 22 ± 6.3 nM), respectively (Table 1).

**Table 1:** Potency and relative agonistic activity (RAA) of typical MTNR ligands in mammals for medaka MTNRs

Ligand	MTNR1a		MTNR1b		MTNR1c		MTNR1a-like	
	Potency (M)	RAA (%)	Potency (M)	RAA (%)	Potency (M)	RAA (%)	Potency (M)	RAA (%)
Melatonin	4.8 ± 2.0 × 10^−10^	100 ± 3.8	1.9 ± 0.49 × 10^−9^	100 ± 4.4	6.2 ± 0.61 × 10^−11^	100 ± 1.8	2.2 ± 0.63 × 10^−8^	100 ± 2.9
2-Iodo melatonin	2.7 ± 1.7 × 10^−11^	95.8 ± 6.9	3.9 ± 0.32 × 10^−11^	101.4 ± 2.8	1.3 ± 0.39 × 10^−11^	111.8 ± 3.2	4.0 ± 1.1 × 10^−10^	69.3 ± 6.8
4P-PDOT	4.7 ± 0.63 × 10^−9^	67.4 ± 6.1	NC	ND	NC	ND	1.2 ± 0.24 × 10^−7^	68.7 ± 6.4
Luzindole	3.3 ± 0.21 × 10^−7^	68.2 ± 3.8	NC	ND	NC	117.6 ± 11.5	NC	83.1 ± 13.4

NC: not calculable (low activity, but its concentration-response curve did not reach maximum levels).

ND: not detected.

### Pharmacological characterization of MTNRs

Pharmacological characterization of the MTNRs was performed with different concentrations of three compounds, known ligands for MTNR1a and/or MTNR1b in mammals. The responses of these analogs varied among the subtypes (Fig. 3, Table 1). While 4P-PDOT and luzindole are known antagonists for MTNRs in mammals, they also exhibited agonistic activity in medaka MTNR1a and MTNR1d. In MTNR1a-expressing cells, the EC_50_ values for 2-Iodomelatonin, 4P-PDOT and luzindole were 0.027, 4.7 and 330 nM, respectively. Their efficacies were weaker than melatonin, as their RAA values were less than 100%. In contrast, 2-Iodomelaonin was potent (EC_50_ = 0.039 nM) against MTNR1b, whereas 4P-PDOT and luzindole had no antagonistic effects. The agnonistic activity of 2-Iodomelatonin was equivalent to that of melatonin for MTNR1b. Concerning MTNR1c, the EC_50_ values for 2-Iodomelatonin and luzindole were 0.013 and greater than 1000 nM, respectively, whereas 4P-PDOT displayed no agonistic effects. Luminescence was decreased in MTNR1c cells treated with 2-Iodomelatonin and luzindole compared with that of melatonin-treated cells, amounting to 112% and 118%, respectively. When *MTNR1d* was transfected in cells, 2-Iodomelatonin, 4P-PDOT and luzindole responded as agonists and their EC_50_ values were 0.40, 120 and >1000 nM, respectively. The maximum inhibition of luciferase transcription of these ligands compared with melatonin were 69.3%, 68.7% and 83.1%, respectively.

**Figure 4:**
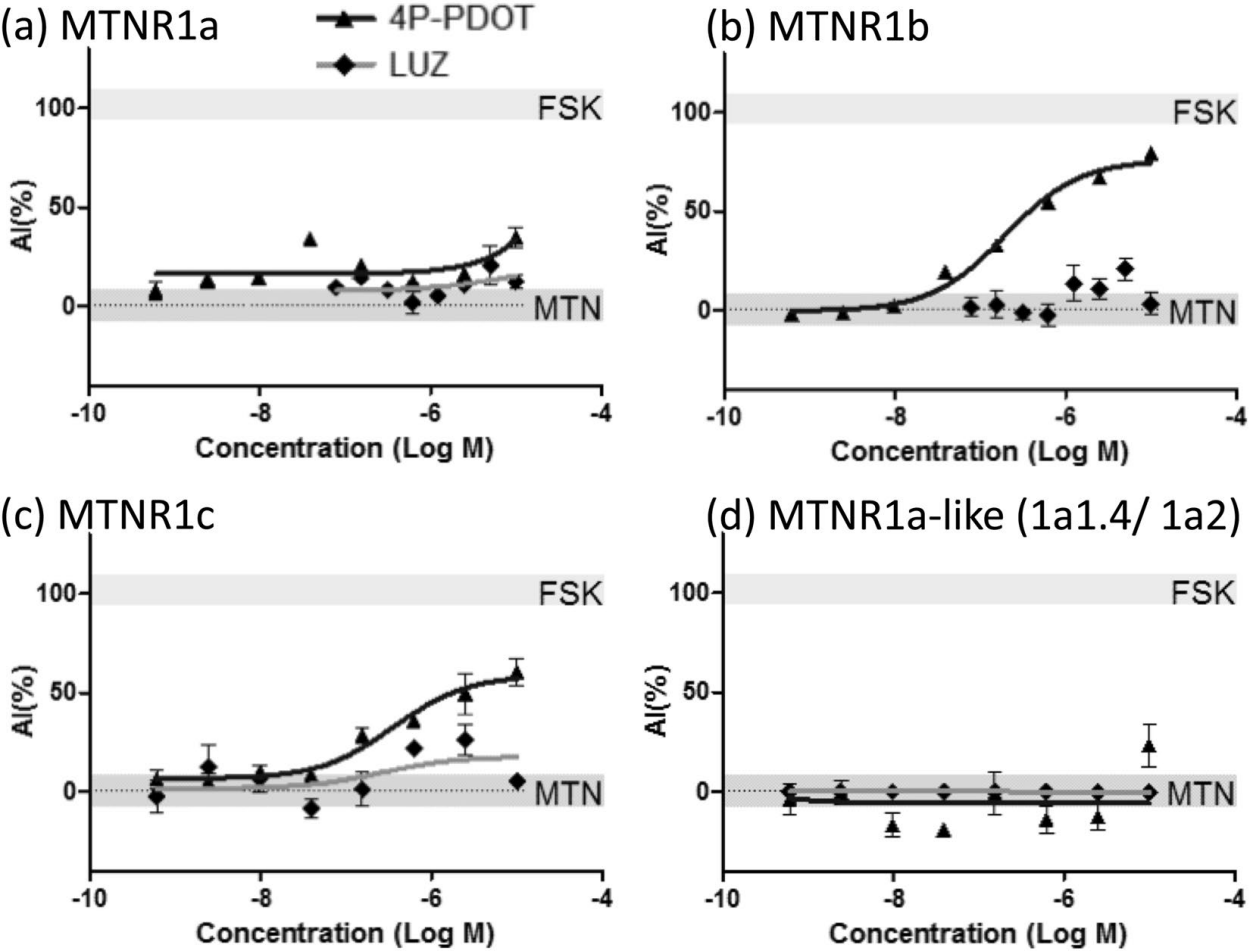
Concentration-response curve of typical MTNR antagonist for medaka MTNRs with EC_90_ melatonin. Y-axis shows antagonistic index (AI) and the x-axis shows the concentration of reagents used for cell treatments. The upper gray box signifies the mean ± standard error of the mean (S.E.M.) value of forskolin (FSK) and the lower gray box represents the average (±S.E.M.) value of the 90% effective concentration of melatonin (MTN). Each point represents the mean of triplicate calculations, and vertical bars represent the S.E.M.

The melatonin-induced inhibition of luciferase transcription activity was antagonized by 4P-PDOT in MTNR1b or MTNR1c expression in cells with 68.3% or 59.8% inhibition, respectively, but luzindole did not exhibit inhibition against melatonin action in any MTNR-expressing cells (Fig. 4, Table 2).

**Table 2:** Potency and antagonistic index (AI) of typical MTNR antagonist in mammals for medaka MTNRs with EC_90_ melatonin.

Ligand	MTNR1a		MTNR1b		MTNR1c		MTNR1a-like	
	Potency (M)	AI (%)	Potency (M)	AI (%)	Potency (M)	AI (%)	Potency (M)	AI (%)
4P-PDOT	NC	ND	1.9 ± 0.22 × 10^−7^	68.3 ± 8.0	NC	59.8 ± 4.9	NC	ND
Luzindole	NC	ND	NC	ND	NC	ND	NC	ND

NC: not calculable (low activity, but its concentration-response curve did not reach maximum levels).

ND: not detected.

### Temporal changes in expression of *mtnr* genes in the brain and eyes

**Figure 5:**
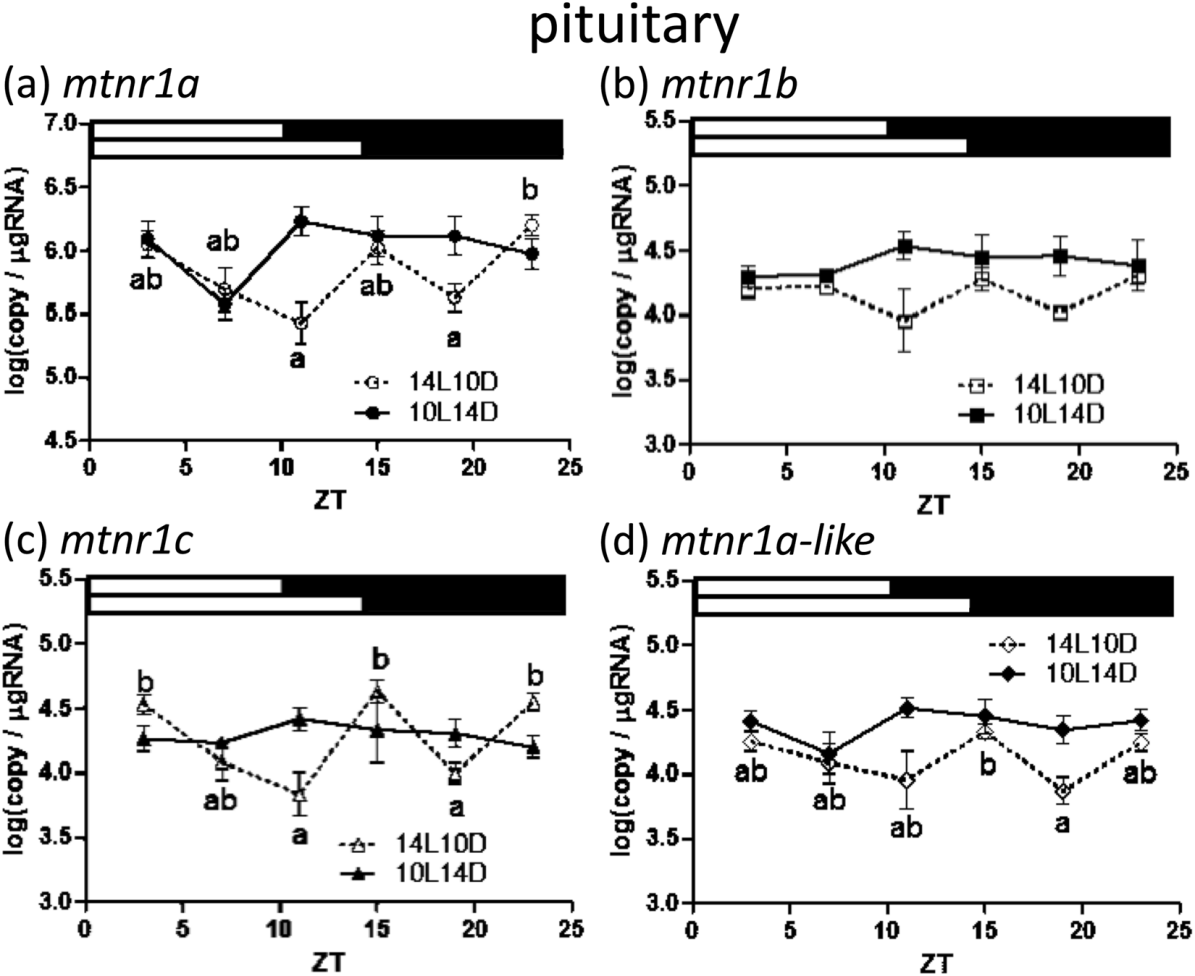
Diurnal expressions of medaka *mtnr1*s in the pituitary under short and long photoperiods as assessed by quantitative real-time (RT)-PCR. The temporal changes in expression of *mtnr1a* (**a**), *mtnr1b* (**b**), *mtnr1c* (**c**) and *mtnr1a-like* (**d**). The sample collection time is indicated as ZT. The white and black bars above each graph represent light and dark periods during 14L10D (upper) and 10L14D (lower). Y-axes represent *mtnr1* expression as copies/μg of total RNA. Data are expressed as mean ± standard error of the mean (S.E.M.) (n = 6). Different letters on the columns indicate group means that are differ statistically when analyzed using one-way ANOVA followed by Tukey’s Multiple Comparison Test (P < 0.05).

**Figure 6:**
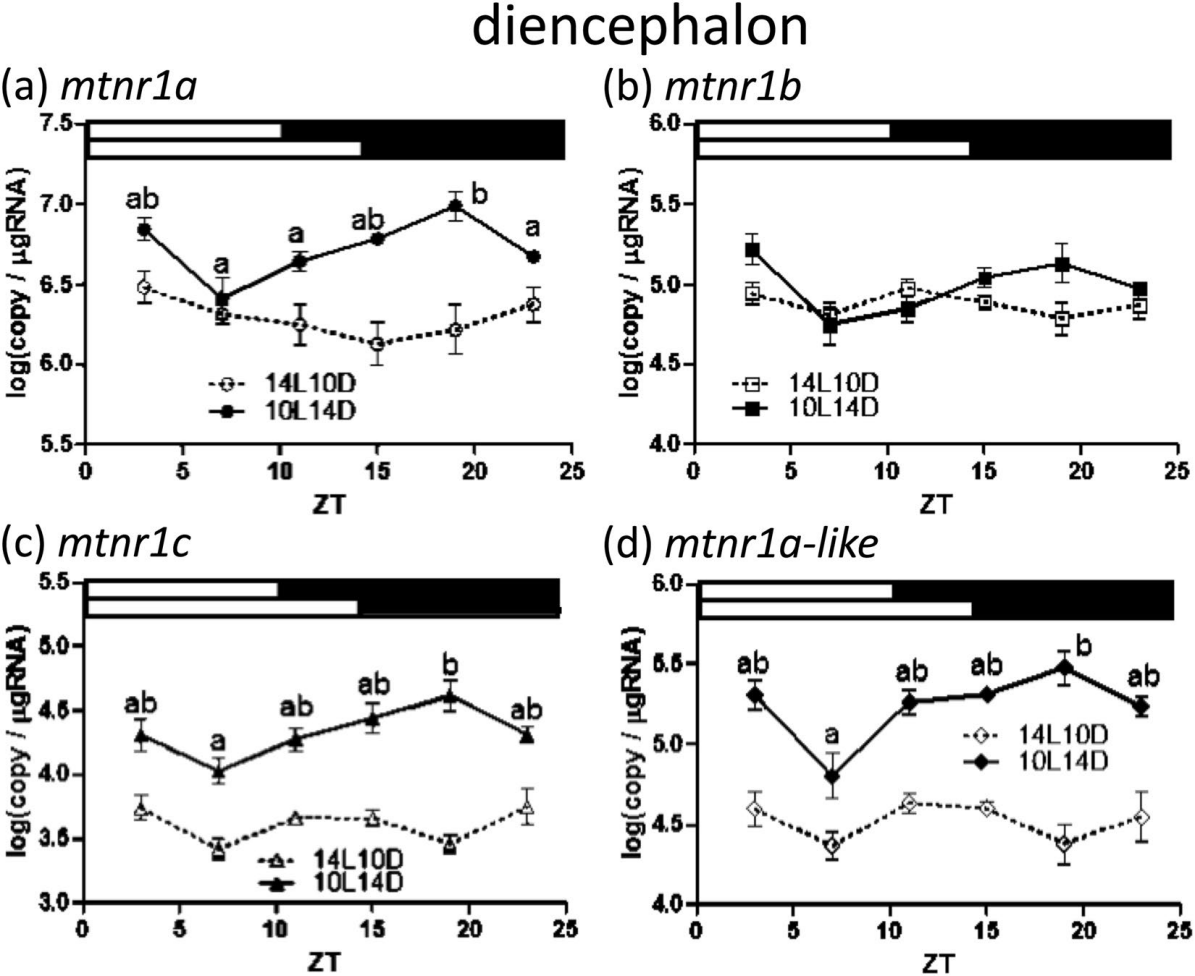
Diurnal expression of medaka *mtnr1*s within diencephalons under short and long photoperiods as assessed by quantitative real-time (RT)-PCR. Temporal changes in expression of *mtnr1a* (**a**), *mtnr1b* (**b**), *mtnr1c* (**c**) and *mtnr1a-like* (**d**). The sample collection time is indicated as ZT. The white and black bars above each graph represent light and dark periods during 14L10D (upper) and 10L14D (lower). Y-axes represent *mtnr1* expression as copies/μg of total RNA. Data are expressed as mean ± standard error of the mean (S.E.M.) (n = 6). Different letters on the columns indicate group means that are differ statistically when analyzed using a one-way ANOVA followed by Tukey’s Multiple Comparison Test (P < 0.05).

**Figure 7:**
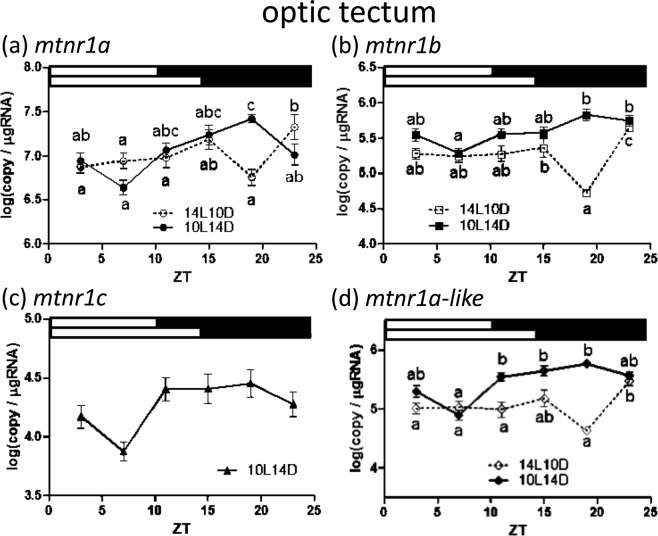
Diurnal expressions of medaka *mtnr1*s in the optic tectum under short and long photoperiods as assessed by quantitative real-time (RT)-PCR. Temporal changes in expression of *mtnr1a* (**a**), *mtnr1b* (**b**), *mtnr1c* (**c**) and *mtnr1a-like* (**d**). The sample collection time is indicated as ZT. The white and black bars above each graph represent light and dark periods during 14L10D (upper) and 10L14D (lower). Y-axes represent *mtnr1* expression as copies/μg total RNA. Data are expressed as mean ± standard error of the mean (S.E.M.) (n = 6). Different letters on the columns indicate group means that are statistically different when analyzed using a one-way ANOVA followed by Tukey’s Multiple Comparison Test (P < 0.05).

**Figure 8:**
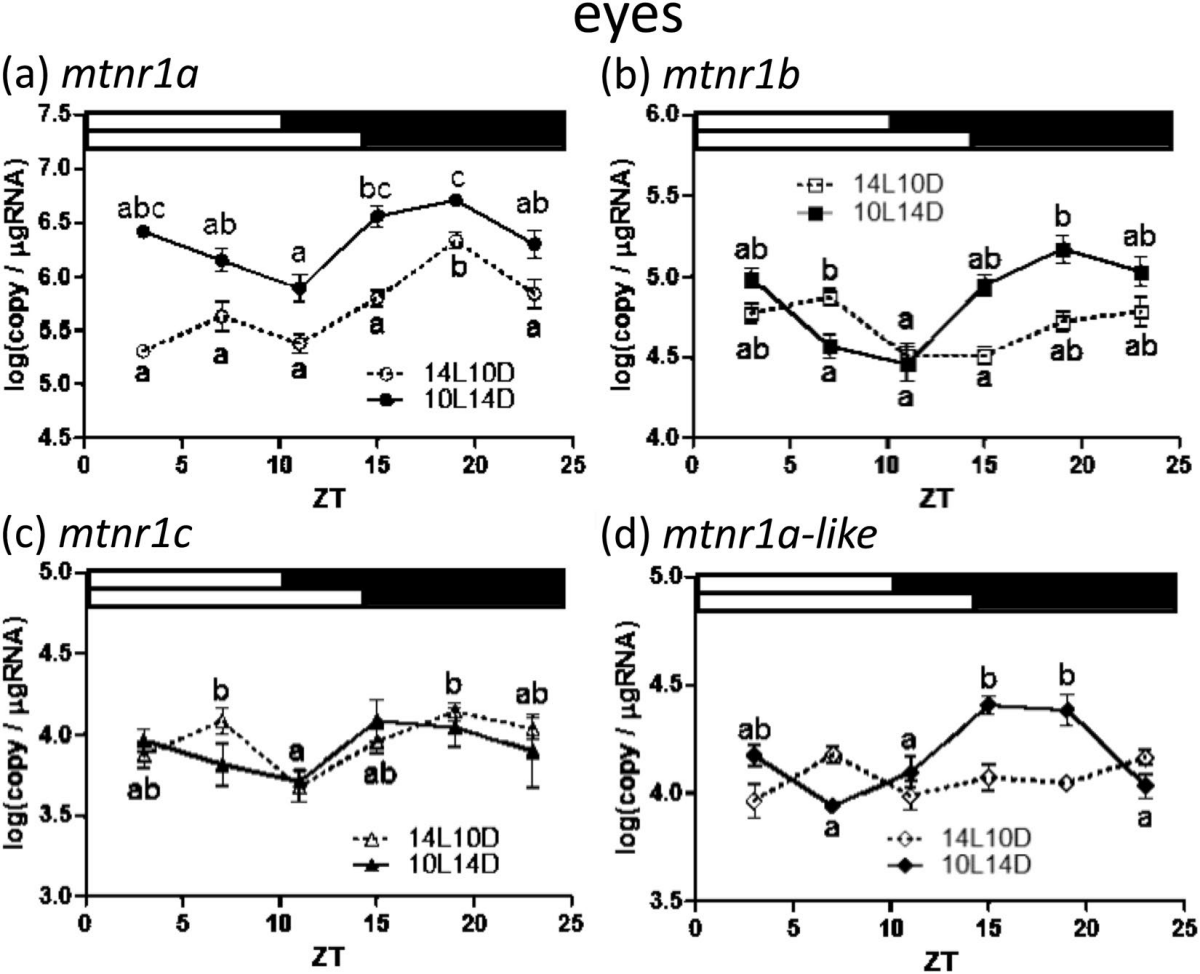
Diurnal expressions of medaka *mtnr1*s in the eyes under short and long photoperiods as assessed by quantitative real-time (RT)-PCR. Temporal changes in expression of *mtnr1a* (**a**), *mtnr1b* (**b**), *mtnr1c* (**c**) and *mtnr1a-like* (**d**). The sample collection time is indicated as ZT. The white and black bars above each graph represent light and dark periods during 14L10D (upper) and 10L14D (lower). Y-axes represent *mtnr* expression as copies/μg total RNA. Data are expressed as mean ± standard error of the mean (S.E.M.) (n = 6). Different letters on the columns indicate group means that are statistically different when analyzed using a one-way ANOVA followed by Tukey’s Multiple Comparison Test (P < 0.05).

The diurnal expression profile for the four *mtnr* genes was examined in the pituitary gland, diencephalon, optic tectum and eyes of the medaka using qPCR. Overwhelmingly, *mtnr1a* was detected at the highest levels in all tested tissues (Figs 5–8). By contrast, the transcript of *mtnr1c* had the lowest expression level of all the *mtnr* genes.

In the pituitary gland, *mtnr1a* mRNA levels fluctuated during the night period; a significantly higher number of transcripts was detected at Zeitgeber time (ZT) 23 versus ZT 11, 19 during 14 h light and 10 h dark cycle (14L10D) (Fig. 5a). No statistically significant difference was observed for *mtnr1b* for either photoperiod (Fig. 5b). Furthermore, *mtnr1c* transcripts were detected at significantly higher levels at ZT 3, 15 and 23 compared to that of ZT 11, 19 during 14L10D (Fig. 5c). In terms of the *mtnr1a-like* gene it was also observed that its mRNA levels fluctuated during nighttime in the pituitary gland during 14L10D, similar to *mtnr1a* and *mtnr1c* (Fig. 5d).

In diencephalons, *mtnr1a*, *mtnr1c* and *mtnr1a-like* expression was elevated during 10 h light and 14 h dark cycle (10L14D) compared to 14L10D (Fig. 6). During 10L14D, the *mtnr1a* mRNA level was significantly higher at ZT 19 than that of ZT 7, 11, and 23 (Fig. 6a). The *mtnr1c* and *mtnr1d* transcripts were also significantly increased at ZT 19 than that of ZT 7 (Fig. 6c,d). No statistically significant difference was observed for *mtnr1b* during either photoperiod (Fig. 6b).

In the optic tectum, *mtnr1a* exhibited a daily variation with a single peak at ZT 19 vs. ZT 3, 7 and 23 during 10L14D, while the expression level at ZT 23 was higher than that of ZT 3, 7, 11 and 19 during 14L10D (Fig. 7a). Significantly higher levels of *mtnr1b* expression were observed at ZT 19, 23 compared to ZT 7 during 10L14D (Fig. 7b). The expression of *mtnr1b* showed daily variation with significantly upregulated levels at ZT 15 and ZT 23 vs. ZT 19 during 14L10D (Fig. 5b). In terms of the *mtnr1a-like* gene, the transcripts were detected at significantly higher levels at ZT 23 compared to that of ZT 3, 7, 11 and 19 during long days (14L10D), whereas the mRNA levels significantly decreased at ZT 7 compared to that of ZT 11, 15 and 19 during short days (10L14D; Fig. 7d). No statistically significant difference was observed for *mtnr1c* during 10L14D, while during the 10L14D *mtnr1c* expression levels fell below measurable limits (Fig. 7c).

In the eyes, a large amount of *mtnr1a* expression was detected during 10L14D compared with 14L10D. The expression level of *mtnr1a* showed a significant change in the eyes with one peak at ZT19 during 14L10D (Fig. 8a). Moreover, *mtnr1a* exhibited a daily variation with high expression at ZT 19 vs. ZT 7, 11 and 23 during 10L14D. Transcription of the *mtnr1b* gene was the second highest after *mtnr1a*, and its expression level at ZT19 was significantly increased compared to ZT 7 and ZT 11 during 10L14D, although the *mtnr1b* mRNA was higher at ZT 7 versus ZT 11 and ZT 15 during 14L10D (Fig. 8b). The mRNA level of *mtnr1c* was significantly elevated at ZT 7, 19 compared to that of ZT 11 during 14L10D (Fig. 8c). In the 10L14D condition, a significantly higher number of *mtnr1a-like* transcripts were detected at ZT 15 and 19 in comparison to ZT 7, 11 and 23 (Fig. 8d).

## Discussion

The present study investigated the following: (1) whether the *MTNR1a-like* gene is the fourth subtype of *MTNR1*, which could be termed *MTNR1d*, (2) if the four subtypes are functional, and (3) if they are actually expressed in cells. In response to the first question, yes, it is popularly considered that *MTNR1a* and *MTNR1b* are present in mammals, and other vertebrates have *MTNR1a*, *MTNR1b* and *MTNR1c*^2^. Additionally, a second *MTNR1a* gene, referred to as *MTNR1a-like*, *MTNR1a2* or *MTNR1a1*.*4*, was identified in teleostean fish^9,11,16^. During recent years, the sequence that shares homology with the *MTNR1a-like* was identified in several reptiles (softshell turtle: ENSPSIG00000008066; anole lizard: ENSACAG00000010003; sea turtle: KB598657.1). Our synteny analysis, which included these *MTNR* subtypes, indicated that some genes located close to the *MTNR* paralogs are conserved among vertebrates and, thus, it would appear that the occurrence of *MTNR1a* and *MTNR1a-like* was not teleost-specific whole genome duplication, but instead a second whole genome duplication. The nodes of a phylogenetic tree exhibiting the divergence between the *MTNR1a*/*MTNR1a-like* and *MTNR1b*/*MTNR1c* groups would suggest that these two groups were divided by the first whole genome duplication and was then bifurcated into four *MTNR*s. Therefore, we would like to emphasize that the fourth *MTNR*, currently called *MTNR1a-like*, should be recognized as *MTNR1d*.

Second, we showed that all four subtypes have the potential to be a receptor for melatonin. In the present study, luminescence in response to the amount of cAMP was decreased by melatonin in a concentration-dependent manner in Hepa-E1 cells transfected with each subtype. This result is consistent with previous reports indicating that the fish melatonin receptor activates the inhibitory G protein, thus leading to decreases in cAMP production by inhibiting the adenylate cyclase^17,18^. This is also evidence for the four subtypes of MTNR in the medaka being functional.

Because comparison of the responses to reagents showed different pharmacological profiles between subtypes, we would like to discuss the relevant points. The order of potency for melatonin was as follows: MTNR1c > MTNR1a > MTNR1b > MTNR1d/MTNR1a-like. MTNR1c has the highest potency with more than three hundred-fold effectiveness compared with MTNR1d/MTNR1a-like. It is possible that these might play different roles depending on the amount of melatonin.

The 2-Iodomelatonin is known as a potent melatonin analog. In a binding assay using 2-[^125^I]iodomelatonin, the pharmacological characteristics of MTNR showed that ligand selectivity varies among melatonin receptor subtypes in many vertebrates^7,17,19–21^. In the medaka, a high potency of this synthetic melatonin-related substance agrees with these previous reports.

Interestingly, luzindole and 4P-PDOT, chemical compounds using as antagonists of the melatonin receptor^22–24^, stimulated both MTNR1a and MTNR1d/MTNR1a-like within the medaka as agonists. In contrast, 4P-PDOT suppressed melatonin-induced inhibition within transcriptional activity of the luciferase gene only in MTNR1b. Thus, 4P-PDOT probably provides an MTNR1b-specific inhibitory effect in the medaka at an organism level.

It was an unexpected result that luzindole did not inhibit any of the medaka MTNR subtypes, although 4P-PDOT effected MTNR1b as an antagonist. Luzindole has been used for inhibition of melatonin receptors in various teleosts^25–28^. Therefore, it is logical that this agent has been selected as an antagonist for piscine MTNRs, as it is a ‘proven’ agent in mammals. To investigate the function of medaka MTNR subtypes and perhaps teleostean MTNRs, it is necessary to develop a new antagonist that specifically binds to each MTNR subtype and inhibits the effect of melatonin for a sufficient period of time.

Last, we showed that the four subtypes were detected by an adequate amount of transcripts and that these have physiologically changed in conditions of light. To demonstrate that these are functional, it is necessary to show their existence as well as potency. In the medaka, there have been no previous reports concerning MTNR expression patterns. In the present study, the melatonin receptor gene transcripts exhibited diurnal variations. Such day-night variations in melatonin receptor expression was also reported in the Senegalese sole (*Solea senegalensis*)^20^. It is possible that expression diurnation in *mtnr*s reflects the roles for entrainment of circadian rhythms to light/dark cycles. Thus, these four MTNR subtypes seem to be functional, and not the products of pseudogenes. However, these transcript levels sometimes remain steady, depending on the tissue, such as the brain, under long-day conditions in our study, which is consistent with a previous report showing significant changes in the expression of *mtnr1a* and *mtnr1b* genes, not in optic tectum but in the retina of goldfish^9^.

Fluctuation in the expression of *mtnr*s in accordance with the photoperiod was also reported in relation to differences in other fish^11,29^. Changes in the expression of *mtnr*s subtypes were observed in association with a photoperiod in medaka, which held its rhythm in the present study. MTNRs may mediate melatonin function in proportion to the amount of hormones since this change was dependent on the length of dark periods. In other words, it is possible that MTNRs in the eyes and diencephalon of the medaka are needed to transduce light information under short-day conditions. Moreover, elevated expression of *mtnr*s during short (10L14D) compared with long days (14L10D) provides evidence that the four subtypes of MTNR within the medaka are functional. Even though *mtnr1a* mRNA was abundant in the eyes as well as brain areas analyzed, other MTNR subtype genes may be expressed locally. Further studies demonstrating tissue localization of MTNRs in more detail are necessary to elucidate the role of each of the melatonin receptors.

## Conclusions

This report showed that vertebrates have four original subtypes of MTNR, and that they are functional as the receptors for melatonin via analysis of molecular phylogeny, pharmacological profiles and tissue-dependent expression patterns. The synteny of the *MTNR1a-like* gene was conserved in vertebrates, including within some reptiles, suggesting that the *MTNR1a* and *MTNR1a-like* genes have diverged not in teleost-specific whole genome duplication but instead second whole genome duplication. Thus, MTNR1a-like should be recognized as a missing MTNR1d in mammals. Pharmacological analysis using a reporter gene assay indicated that the four MTNR subtypes of the medaka are functional, with differing potencies for melatonin. All the subtypes were expressed to some level in the brain and eyes. MTNR1a mRNA was highly abundant in the medaka brain and eyes, with diurnal rhythmicity increasing at night. Moreover, the expression level of *mtnr1a* was more enhanced during the non-reproductive photoperiod versus the reproductive photoperiod. These results suggest that the roles of MTNRs are associated with photoperiod-dependent physiological action in teleost fish. Our results also indicate that vertebrates have essentially four functional MTNR subtypes, although some organisms have lost one or two subtypes, including mammals.

## Materials and Methods

### Assessments of conserved synteny

The *MTNR* genes of 11 vertebrate species were compared to syntenic regions (Table 3) near each medaka subtype using Genomicus^30^, including: six teleosts, the medaka (*Oryzias latipes*), amazon molly (*Poecilia formosa*), platyfish (*Xiphophorus maculatus*), tilapia (*Oreochromis niloticus*), stickleback (*Gasterosteus aculeatus*), and tetraodon (*Tetraodon nigroviridis*); one holostean fish, the spotted gar (*Lepisosteus oculatus*); and four tetrapods, the soft-shelled turtle (*Pelodiscus sinensis*), anole lizard (*Anolis carolinensis*), chicken (*Gallus gallus*) and human (*Homo sapiens*). When the *mtnr* gene was not found within the syntenic region of a species, we also used the program Genscan^31^ to confirm whether the *mtnr* was lost or unannotated.

**Table 3:** Chromosomes with *mtnr* genes.

	medaka	amazon molly	platyfish	tilapia
*mtnr1a*	Chr:1	Chr:KI519642.1	Chr:5	Chr:GL831212.1
*mtnr1b*	Chr:14	Chr:KI519613.1	Chr:11	Chr:GL831183.1
*mtnr1c*	Chr:10	Chr:KI519738.1	Chr:23	Chr:GL831174.1
*mtnr1d*	Chr:10	Chr:KI519678.1	Chr:23	Chr:GL831190.1
	**spotted gar**	**stikleback**	**tetraodon**	**softshell tuetle**
*mtnr1a*	Chr: LG4	Chr:groupIX	Chr:18	Chr:JH210278.1
*mtnr1b*	Chr:LG3	Chr:groupVII	Chr:7	Chr:JH205573.1
*mtnr1c*	Chr:LG7	Chr:groupIV	Chr:1	Chr:JH224646.1
*mtnr1d*	Chr:LG6	Chr:groupIV	Chr:1	Chr:JH224652.1
	**anole lizard**	**chicken**	**human**	
*mtnr1a*	Chr:5	Chr:4	Chr:4	
*mtnr1b*	Chr:3	Chr:1	Chr:11	
*mtnr1c*	Chr:GL343310.1	Chr:4	Chr:X	
*mtnr1d*	Chr:2	Chr:13	Chr:5	

### Phylogenetic analysis

The protein sequences of the MTNRs were retrieved from the Ensembl database and National Center for Biotechnology Information (NCBI). Amino acid sequence IDs were as follows: human 1a: NP_005949.1, 1b: NP_005950.1; chicken 1a: NP_990693.1, 1b: NP_001280032.1, 1c: NP_990692.1; sea turtle (*Chelonia mydas*) 1a1: EMP24711.1, 1a3: EMP33109.1; soft-shelled turtle 1a: ENSPSIT00000001974.1, 1b: ENSPSIT00000001684.1, 1c: ENSPSIT00000000237.1, 1a-like: ENSPSIT00000008874.1; anole lizard 1a: ENSACAT00000007042.2, 1b: ENSACAT00000014670.2, 1c: NSACAT00000013281.2, 1a-like: ENSACAT00000009998.3; spotted gar 1b: ENSLOCT00000008866.1, 1c: ENSLOCT00000018184.1, 1a-like: ENSLOCT00000014391.1; medaka 1a: XP_004066003.1, 1b: BAS31074.1, 1c: BAQ59091.1, 1a-like: XP_004073660.1, DRD2: XP_004075461.1; stickleback 1a: ENSGACT00000022338.1, 1b: ENSGACT00000027283.1, 1c: ENSGACT00000024260.1, 1a-like: ENSGACT00000022258.1; tilapia 1a: ENSONIT00000012798.1, 1b: ENSONIT00000005979.1, 1c: ENSONIT00000022473.1, 1a-like: ENSONIT00000005358.1; tetraodon 1a: ENSTNIT00000010105.1, 1b: ENSTNIT00000003970.1, 1c: ENSTNIG00000008982, 1a-like: ENSTNIT00000003673.1. Amino acid sequences of the MTNRs were aligned using ClustalW and displayed in MEGA 7.0.14 (Molecular Evolutionary Genetics Analysis, ver. 7.0.14), and then manually adjusted. Phylogenetic trees were constructed with the MEGA 7.0.14 software using the neighbor-joining method^32^ with branch support values estimated using bootstrap analysis with 1000 replications.

### Experimental animals

All of the animal experiments described below were conducted in compliance with institutional guidelines and were approved by the Animal Experiment Committee of Nagahama Institute of Bio-Science and Technology. Mature medaka fish of the orange-red variety (body length = 24–29 mm) were acclimated to conditions of 26–27 °C on 14L10D or 10L14D for at least 30 days prior to sampling. All fish were anaesthetized in aqueous solution of 0.01% ethyl *p*-aminobenzoate buffered with 0.01% sodium hydrogen carbonate before euthanasia. For expression analysis the pituitary gland, diencephalon, optic tectum and eyes were collected every fourth hour [ZT 3, 7, 11, 15, 19, 23 h after “light on”]. Samples were immersed in Sepasol RNA I Super G (Nacalai Tesque) and kept at −80 °C until use.

### Development of medaka *mtnr* gene expression construct, transfection into Hepa-E1 cells, and reporter gene assay

Expression vectors for medaka *mtnr* genes, (pcDNA-OlMTNR1a, pcDNA-OlMTNR1b, pcDNA-OlMTNR1c and pcDNA-OlMTNR1a-like) were constructed by PCR amplification of the entire protein coding region, using primers which introduced in-frame Kozak sequence^33^ at the 5′ end. A cyclic adenosine monophosphate (cAMP)-regulated reporter vector containing the luciferase gene under the control of a cAMP response element upstream of the TATA-like promoter region from the herpes simplex virus thymidine kinase promoter, named pCRE-luc, originated from the Pathway Profiling Luciferase System (Clontech). Hepa-E1 cells, i.e. epithelial-like Nile tilapia hepatocytes, were obtained from the Institute of Physical and Chemical Research (RIKEN) cell bank (Tsukuba, Japan). For MTNR1a, MTNR1b and MTNR1d, stable transfections were performed as described below. Hepa-E1 cells were seeded in 9 cm dishes prior to transfection. Both plasmids, pCRE-luc as well as expression vectors for medaka *mtnrs* were linearized by PCR and transfected with X-tremeGENE 9 (Roche) according to the manufacturer’s instructions. Stably transfected cells were selected with G418. After selection, each colony was collected by pipetting. Sensitivity for cAMP in each clone was tested by forskolin treatment after seeding into 96-well plates with E-RDF medium (Kyokuto, Tokyo, Japan) and supplemented with 5% dextran-charcoal treated fetal bovine serum. After 24 h the cells were collected, and cellular luciferase activities were measured by luminescence assay with the Steady-Glo Assay System Kit (Promega) according to the manufacturer’s instructions. Forskolin-sensitive cell lines derived from co-transfection of pCRE-luc and pcDNA3.1(+) were developed as control lines and named null-CRE-luc. Forskolin-sensitive cell lines derived from co-transfection of pCRE-luc and each pcDNA-OlMTNR1 were selected for by sensitivity for melatonin.

For MTNR1c, transient transfection was performed as outlined below. The MTNR1c experiment was performed using Hepa-E1 cells in 9 cm dishes, which were transiently transfected by pcDNA-OlMTNR1c (500 ng) and pCRE-luc (5000 ng) by TransIT-X2 Dynamic Delivery System (Mirus) and incubated 24 h before seeding, following the manufacturer’s instructions. Cells derived from a chosen clone or MRNR1c-tranfected cells were seeded at 10^4^ cells per well in 96-well luminometer plates. Then, cells were treated with test chemicals in triplicate at four-fold serial dilutions in the presence or absence of melatonin with 6.25 × 10^−7^ M forskolin. For testing the antagonistic effect, reagents were mixed with melatonin at 90% effective concentration (EC_90_). Negative control wells were dosed with media plus 0.1% ethanol. Positive control wells were dosed with 1 μM melatonin. After 24 h, luciferase activities were measured as described above. Concentration-response curves were fitted and the EC_50_ or IC_50_ were calculated using sigmoidal fit in GraphPad Prism5. The relative agonistic activity (RAA) of each reagent was expressed as a percentage of the maximal activity for the reporter induction to that of melatonin. The antagonistic index (AI) of each reagent was expressed as a percentage of the maximal inhibition of the reagent. Maximal inhibition was calculated as the quotient of the subtraction of the luminescence for melatonin from the maximal luminescence for the reagent divided by the maximal inhibition of melatonin. When the reagent inhibits the transactivity to the same level of that of the vehicle control (forskolin), the AI equals 100%.

### Quantitative Real-time PCR

Total RNA from each tissue was extracted using Sepasol RNA I Super G (Nacalai Tesque) according to the manufacturer’s instruction. Total RNA was reverse-transcribed and genomic DNA was removed using ReverTra Ace qPCR RT Master Mix with gDNA Remover (TOYOBO). Quantitative Real-time PCR was conducted with SsoAdvanced Universal SYBR Green Supermix (BIO-RAD) using the MiniOpticon real-time PCR system (BIO-RAD). The primer set for *mtnr1a* (XM_004065955.3) was 5′-AGCGTATACAATCGCAGTGGTG-3′ (forward) and 5′-TTGGTCGGTTGTCTGGTTTG-3′ (reverse). Primers for *mtnr1b* (LC032111.1) were 5′-CGGCGTAAAGTAAAGACTGAAGAAA-3′ (forward) and 5′-CAGATGGCGAACAGCACAA-3′ (reverse). Primers for *mtnr1c* (LC033812.1) were 5′-GCTGCCTCAATGCCATCATA-3′ (forward) and 5′-CGGCACGCAAAGAGCA-3′ (reverse). Primers for *mtnr1a-like* (XM_004073612.3) were 5′-GGCACCTTCCGCAACTGA-3′ (forward) and 5′-CCTGTCGGCGTGGAGTTG-3′ (reverse). These primers were designed using Primer3 ver.0.4.0 (1, 2). The conditions for PCR were as follows: initial denaturation at 98 °C for 2 min, 40 amplification cycles with denaturation at 98 °C for 2 s, and annealing at 60 °C for 5 s. Standards for the *mtnr1a* plasmid were constructed with a 10-fold serial dilution series ranging from 4.79 × 10^3^ to 4.79 × 10^10^ copies per µL. For *mtnr1b*, *mtnr1c* and *mtnr1d*, the dilution series ranged from 4.73, 4,79 and 4.77 × 10^3^ to 4.73, 4,79 and 4.77 × 10^10^ copy/µL, respectively. The amplification efficiency of the standard curves was between 91 and 107%. Measured values were normalized for the amount of total RNA and presented as copy/μg of total RNA. For statistical evaluation of results and significance testing of group differences, data was analyzed by one-way ANOVA followed by Tukey’s Multiple Comparison Test using GraphPad Prism5 (GraphPad Software, San Diego, CA).

## Data Availability

The data supporting the conclusions of this article are included within the article and its additional files.
